# Effects of Wheat Straw Incorporation on the Availability of Soil Nutrients and Enzyme Activities in Semiarid Areas

**DOI:** 10.1371/journal.pone.0120994

**Published:** 2015-04-16

**Authors:** Ting Wei, Peng Zhang, Ke Wang, Ruixia Ding, Baoping Yang, Junfeng Nie, Zhikuan Jia, Qingfang Han

**Affiliations:** 1 The Chinese Institute of Water-saving Agriculture, Northwest A&F University, Yangling, 712100, Shaanxi, China; 2 Key Laboratory of Crop Physi-ecology and Tillage Science in Northwestern Loess Plateau, Ministry of Agriculture, Northwest A&F University, Yangling, 712100, Shaanxi, China; Genetics and Microbiology Research Group, SPAIN

## Abstract

Soil infertility is the main barrier to dryland agricultural production in China. To provide a basis for the establishment of a soil amelioration technical system for rainfed fields in the semiarid area of northwest China, we conducted a four—year (2007–2011) field experiment to determine the effects of wheat straw incorporation on the arid soil nutrient levels of cropland cultivated with winter wheat after different straw incorporation levels. Three wheat straw incorporation levels were tested (H: 9000 kg hm^-2^, M: 6000 kg hm^-2^, and L: 3000 kg hm^-2^) and no straw incorporation was used as the control (CK). The levels of soil nutrients, soil organic carbon (SOC), soil labile organic carbon (LOC), and enzyme activities were analyzed each year after the wheat harvest. After straw incorporation for four years, the results showed that variable straw amounts had different effects on the soil fertility indices, where treatment H had the greatest effect. Compared with CK, the average soil available N, available P, available K, SOC, and LOC levels were higher in the 0–40 cm soil layers after straw incorporation treatments, i.e., 9.1–30.5%, 9.8–69.5%, 10.3–27.3%, 0.7–23.4%, and 44.4–49.4% higher, respectively. On average, the urease, phosphatase, and invertase levels in the 0–40 cm soil layers were 24.4–31.3%, 9.9–36.4%, and 42.9–65.3% higher, respectively. Higher yields coupled with higher nutrient contents were achieved with H, M and L compared with CK, where these treatments increased the crop yields by 26.75%, 21.51%, and 7.15%, respectively.

## Introduction

In recent years, the increasingly severe food crisis means that producing sufficient food to sustain the huge population of China has become a major priority of the government. Chemical fertilizer application is one of the most common agricultural management practices and it has made a significant contribution to crop yield increases [1]. However, long-term excessive fertilization can lead to many problems, such as deterioration in the soil [2] and groundwater quality, marked nutritional disorders in farmland, and environmental damage from nonpoint source pollution [3]. Thus, many researchers have proposed the replacement of chemical fertilizers with organic fertilizers [4,5].

Organic fertilizers contain high levels of specific nutrients and they generally have a high organic matter content with a variety of micronutrients [6]. As one of the main natural organic fertilizer sources, ca 600–800 million tons crop straw are produced each year in China. Many studies have shown that crop straw is rich in organic material and soil nutrients [7,8], the addition of crop residues to cultivated soils helps to improve the soil quality and productivity via its favorable effects on soil properties [9].

Recently, soil quality has gained attention as a result of environmental issues related to soil degradation and production sustainability under different farming systems [10]. It has been considered by previous researches that the concentrations of soil nutrients (e.g., organic C, N, P, and K) are good indicators of soil quality and productivity because of their favorable effects on the physical, chemical, and biological properties of soil [11]. Soil enzymes, which have been shown to be related to microbial activity, catalyse reactions in soils that are important in nutrient cycling. And the soil organic carbon (SOC) is known to be a key factor in soil quality of semiarid soils. Improving SOC levels helps to maintain nutrient availability and agricultural sustainability. The long-term decline in soil fertility is a result of the partial or complete removal of aboveground biomass, so crop straw can be incorporated into soil to provide readily available nutrients and to minimize the loss of crop straw [2]. Most current studies have shown that straw incorporation can replenish the soil organic matter by enhancing carbon inputs [12], which has a positive effect on the accumulation of nutrients and it improves the nutrient utilization efficiency [1,13,14]. Some has also been reported that straw incorporation has significant roles in improving the activity levels of soil enzymes and microbial biomass communities [15]. Thus, straw incorporation could become an important method to facilitate sustained cost reductions and efficiency improvements, thereby contributing to the protection of farmland eco-environments.

As a typical dryland farming region located in north-central Shaanxi Province, Weibei Highland is known as “the second granary of Shaanxi”, which hold a pivotal status in ensuring food supply safety of Shaanxi and China. Soil infertility [16], and water deficiency [17] are the major factors that limited crop growth in this area. Most previous studies have focused on the comparison between straw incorporation mode and different types of conservation tillage systems [18,19], and effects of straw incorporation on SOC levels [7], soil structure [9], and enzyme activities [20]. However, the detailed studies on the change of labile organic carbon (LOC) contents and enzyme activities under different straw incorporation rates in semiarid areas, particularly in Weibei areas of China has been rarely reported. Thus, we conducted a straw incorporation experiments to determine the effects of different straw incorporation rates on the soil nutrients and soil enzyme activity levels in Weibei Highland, in order to serve as reference for regional agriculture development.

## Materials and methods

### 2.1 Ethics Statements

The study was carried out on the private land, we rent the farmland from the local farmers, and contracts and deeds are signed. No specific permissions were required in this area to run the experiment as the study sites are farming area without any protection zone, and the farming activities won't hurt the local animals. And we only plant the grain crop in the field, so the field studies did not involve endangered or protected species.

### 2.2 Site description

A four-year field experiment was conducted between 2007 and 2011 using land under winter wheat cultivation at the Ganjing Research Station of Northwest A&F University, Heyang, Shaanxi, China (35°24′N, 110°17′E; 850 m altitude). This region was characterized by low and erratic rainfall, with occasional droughts at different wheat growth stages and has a warm temperate and semi-humid drought climate with an annual precipitation of 572 mm (most of which occurred from July to September), annual evaporation of 1833 mm, and a mean annual temperature of 9–10°C. The soil was Lou soil (Eum-orthic Anthrosol classfied using Chinese Soil Taxonomy, a dark loessial soil with 26.8% sand, 41.9% silt, and 21.3% clay), pH = 8.2 and C/N in 0–60 cm was 11.63. Prior to the experiment, winter wheat was be cultivated for three years with recommended mineral fertilization to ensure no apparent spatial difference in soil physical and chemical properties in the plowing zone over the field. Above ground biomass was removed from the fields after the harvest. The nutrient contents of the 0–60 cm soil layers and the wheat straw used for different treatments are shown in Table 1.

**Table 1:** Organic carbon and nutrient contents of the trial soil and wheat straw in 2007.

Nutrients	The trial soil			
	0–20 cm	20–40 cm	40–60 cm	Wheat straw
Organic carbon (g kg^-1^)	14.04	11.60	8.25	669.15
Total—N (g kg^-1^)	0.69	0.55	0.44	9.83
Total—P (g kg^-1^)	0.66	0.54	0.37	0.37
Total—K (g kg^-1^)	9.34	10.17	10.81	37.81
Available—N (mg kg^-1^)	54	37	28	—
Available—P (mg kg^-1^)	18	8	4	0
Available—K (mg kg^-1^)	142	101	84	15

### 2.3 Experimental design

The experiment was designed as a randomized block with three replicates. The area of each plot was 26.4 m^2^ (8.8 × 3 m). Four treatments were initiated during 2007–2011: (i) no wheat straw incorporation (CK); (ii) incorporation of wheat straw at a low rate of 3000 kg hm^-2^ (L); (iii) incorporation of wheat straw at a medium rate of 6000 kg hm^-2^ (M); (iv) incorporation of wheat straw at a high rate of 9000 kg hm^-2^ (H).

### 2.4 Fertilizer and crop management

During each year, the wheat straw was mixed with soil to a depth of 0–25 cm, where it was turned over into soil after the previous winter wheat harvest in June. The wheat straw in our trail come from two main sources: residues produced by the experiment itself and straw (from the same wheat variety as our trial) from nearby farmland. All of the straw was chopped into pieces that measured approximately 5 cm and mixed manually with the top of soil in the field.

Chemical fertilizers were applied separately ten days before sowing at rates of: N = 150 kg hm^-2^, P_2_O_5_ = 120 kg hm^-2^, and K_2_O = 90 kg hm^-2^. The winter wheat (cv. Jinmai 47) was planted at a rate of 150 kg hm^-2^ on 20 September 2007, 22 September 2008, 25 September 2009, 21 September 2010, using an Amozone NT 250 drill with chisel-typle openers and depth-controlling press wheels at a row spacing of 20 cm. For each crop cycle, manual weeding was undertaken as required during the experiment period. Wheat was harvested on 17 June 2008, 14 June 2009, 15 June 2010, and 18 June 2011. No irrigation was provided.

### 2.5 Sampling and measurement

The rainfall data were recorded using a standard weather station located at the experimental site. Monthly precipitation distributions during the experimental period are shown in Table 2.

**Table 2:** Distribution of mean monthly precipitation (mm) at the experimental site during 2007–2011.

Year	Fallow period			Wheat growing season									Annual
	July	Aug.	Sep.	Oct.	Nov.	Dec.	Jan.	Feb.	Mar.	Apr.	May	June	
2007–2008	197	83	56	48	2	7	29	8	13	32	24	106	604
2008–2009	54	124	65	15	14	1	11	23	20	13	137	47	524
2009–2010	47	97	52	25	38	2	9	21	11	40	44	57	443
2010–2011	75	77	41	45	4	2	3	13	15	29	36	25	363

Soil samples were collected from the 0–20, 20–40, and 40–60 cm layers of all plots immediately after harvest (i.e., before straw incorporation) during June each year. In each plot, soil samples were collected from five points and mixed to form one composite sample. A soil sample that weighed approximately 500 g was subsampled from the composite sample by quartering. Approximately 200 g of this soil subsample was then used to analyze the nutrient contents, and the remainder was sieved, mixed, and stored immediately at 4°C, before analyzing its enzymatic activity.

Available nitrogen (AN) was extracted with 1 M KCL and analyzed using the cadmium reduction method [21]. Available phosphorus (AP) was extracted with a 0.5 M NaHCO_3_ solution, which was adjusted to pH 8.5 [22]. Available potassium (AK) was extracted with neutral 1N NH_4_OAc [23]. Soil organic carbon (SOC) was determined by the K_2_Cr_2_O_7_–H_2_SO_4_ digestion method [24].

Labile organic carbon (LOC) was determined by the method of 333 mM KMnO_4_ oxidation [25], using soil sample weights containing about 15 mg C and 25 ml 333 mM KMnO_4_ shaken for 1.0 h. After centrifugation (5 min at 2000 rpm), absorbances of the diluted samples (1:250 with deionized water) were determined at 565 nm. The amount of carbon oxidized was calculated from the change in the concentration of KMnO_4_ when compared with the blank samples that contained no soil. The Labile C = the C oxidized by 333 mM KMn0_4_.

The urease and alkaline phosphatase activities were determined according to the method of Tabatabai [26]. The invertase activity was determined by colorimetric analysis using 3,5-dinitrosalicylic acid [27]. All of the enzyme activity values were calculated based on the oven-dried (105°C) weight of the soil.

The grain yield was determined at a water content of 12% after manually harvesting the three central rows with a length of 5-m taken randomly from each plot.

### 2.6 Statistical analysis

The mean values were calculated for each parameter and the effects of incorporating various amounts of straw were evaluated by analysis of variance (ANOVA) using SAS 6.2 (SAS Institute Inc., Cary, NC). Duncan’s new multiple range test was performed to compare the mean values when the F-test indicated statistical significance at *P* < 0.05.

## Results

### 3.1 Soil available nutrients

Table 3 shows the AN contents of different soil layers after the winter wheat harvest during 2008–2011. The four-year straw incorporation led to an increase in the AN contents of the 0–40 cm layers, which depended on the amount of straw incorporation. Compared with the control, the AN contents in 2011 were 32.6% and 24.8% significantly higher (P < 0.05) with the H and M treatments, respectively, there were no significant differences between CK and L

**Table 3:** Soil available nutrients content under different wheat straw incorporation treatments in 0–20 and 20–40 cm soil layers from 2008 to 2011.

Years	Treatments	AN (mg kg^-1^)		AP (mg kg^-1^)		AK (mg kg^-1^)	
		0–20 cm	20–40 cm	0–20 cm	20–40 cm	0–20 cm	20–40 cm
2008	H	58±0.6^1^a^2^	42±0.8a	21±0.6a	9.1±0.8a	163±2.9a	109±2.3a
	M	57±0.8a	41±0.8a	18±0.6a	8.7±0.3a	165±1.7a	103±1.9a
	L	53±0.8ab	38±0.5b	16±0.5ab	6.7±0.8ab	152±1.8b	101±2.3a
	CK	51±0.7b	33±0.8c	14±0.2b	5.8±0.6b	136±1.5b	94±1.4a
2009	H	61±0.7a	43±0.6a	22±0.6a	9.4±0.6a	165±1.9a	112±1.7a
	M	59±1.1ab	41±0.8a	19±0.7b	9.0±0.9ab	169±2.1a	106±1.6ab
	L	53±1.0bc	39±0.4b	16±1.0bc	6.7±0.5bc	152±1.5b	103±2.4abc
	CK	51±1.1c	32±0.4b	15±0.5c	5.9±0.7c	133±1.6c	95±1.5c
2010	H	62±0.7a	44±0.5a	24±0.2a	10.6±0.1a	171±2.3a	119±1.8a
	M	59±0.4ab	42±1.1a	21±0.6ab	9.7±0.4a	169±2.7a	111±1.1a
	L	53±1.3c	39±0.6a	18±0.8bc	6.9±0.3b	154±1.7ab	109±2.5ab
	CK	51±1.1c	31±0.8b	16±0.4c	6.2±0.9b	131±1.4bc	98±1.9c
2011	H	66±0.5a	45±0.6a	28±0.3a	12.3±0.7a	180±3.1a	128±1.6a
	M	62±0.7ab	42±0.7ab	24±0.5b	10.4±0.2ab	174±2.3a	121±1.4ab
	L	55±0.4c	39±0.3b	20±0.3c	7.3±0.5bc	160±1.8ab	117±2.1ab
	CK	53±0.8c	32±0.7c	17±0.7c	6.4±0.8c	138±2.2b	104±2.2b

Note: CK, no wheat straw incorporation; L, incorporation of straw at a low rate of 3 000 kg hm^-2^ wheat straw; M, incorporation of straw at a medium rate of 6 000 kg hm^-2^ wheat straw; H, incorporation of straw at a high rate of 9 000 kg hm^-2^ wheat straw. AN, available nitrogen; AP, available phosphorus; AK, available potassium

^1^ Means ± standard deviations

^2^ Values within a column for the same year followed by different letters are significantly different (*P*<0.05)

The variation trend of AP contents in the 0–40 cm layers were similar to that of AN contents. Compared with CK, H treatment resulted in higher AP contents (P < 0.05), and the increase were in the range of 44.4–61.4% throughout the straw incorporation experiment, followed by M treatment with an increase between 25.9% to 39.9% higher than CK, the differences were also significant.

Similar to the changing trend of AP contents, the AK contents of the 0–20 cm soil layers were significantly higher with the H and M treatments than the CK (P < 0.05), i.e., the average increases were 26.6% and 26.1% higher throughout the study period with H and M, respectively. The AK contents in 20–40 cm soil layers were influenced by wheat straw incorporation treatments as well, but the impact degree were less than that in 0–20 cm soil layers. Throughout the straw incorporation experiment, the average increase of the AK contents with H in 20–40 cm soil layers were 19.1% significantly higher than those with CK.

### 3.2 Soil carbon contents

Table 4 shows that compared with CK, all wheat straw incorporation treatments resulted in higher SOC contents in the 0–40 cm soil layers throughout the four experimental years, but there were no obvious interannual variation. And the average increase of SOC contents with H, M and L treatments in 2008–2011 were 21.5%, 18.2% and 3.1% higher than CK treatment, only the H and M treatments produced the significant differences (*P* < 0.05).

**Table 4:** Soil carbon content under different wheat straw incorporation treatments in 0–20 and 20–40 cm soil layers from 2008 to 2011.

Years	Treatments	Organic carbon (g kg^-1^)		Labile organic carbon (g kg^-1^)	
		0–20 cm	20–40 cm	0–20 cm	20–40 cm
2008	H	17±0.7^1^a^2^	11±0.6a	—	—
	M	17±0.2ab	11±0.8a	—	—
	L	15±0.5b	9±1.0b	—	—
	CK	15±0.9b	9±0.3b	—	—
2009	H	17±0.3a	11±1.1a	—	—
	M	17±0.6ab	11±0.4a	—	—
	L	16±0.5bc	9±0.7b	—	—
	CK	15±0.4c	9±0.4b	—	—
2010	H	17±0.4a	12±0.3a	6.5±0.3ab	4.1±0.1ab
	M	17±0.3ab	12±1.0a	6.5±0.3ab	4.1±0.3ab
	L	16±0.3bc	9±0.6b	5.8±0.2b	3.8±0.1b
	CK	15±0.3c	9±0.4b	3.5±0.2c	2.9±0.9c
2011	H	18±0.2a	14±0.8a	6.6±0.2a	4.4±0.1a
	M	17±0.2a	13±0.5b	6.5±0.2a	4.3±0.1a
	L	16±0.1b	11±03bc	5.7±0.2b	3.9±0.3a
	CK	15±0.2b	10±0.5c	3.7±0.3c	3.1±0.2b

Note: CK, no straw incorporation; L, incorporation of straw at a low rate of 3 000 kg hm^-2^ wheat straw; M, incorporation of straw at a medium rate of 6 000 kg hm^-2^ wheat straw; H, incorporation of straw at a high rate of 9 000 kg hm^-2^ wheat straw.

^1^ Means ± standard deviations

^2^ Values within a column for the same year followed by different letters are significantly different (*P*<0.05)

Table 4 shows the LOC contents during 2010 and 2011. In the 0–40 cm soil layers, the LOC contents with the different treatments during 2010 were ranked in the order: M > H > L > CK, whereas in 2011, the order was: H > M > L > CK. In the 0–20 cm layers, the LOC contents were 82.2% and 78.1% higher in 2010 and 2011, respectively, with the H treatment than CK, 82.5% and 76.4% higher with M, and 64.2% and 55.6% higher with L (*P* < 0.05). In the 20–40 cm layers, the LOC contents with all the incorporation treatments were 32.2–44.0% higher compared with CK in 2010 and 27.6–43.7% higher in 2011.

### 3.3 Soil enzyme activity

#### 3.3.1 Soil urease activity

**Fig 1:**
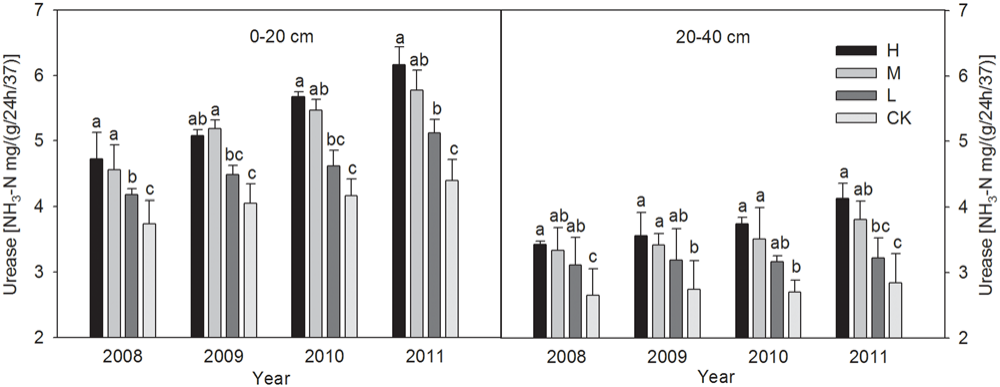
The changes of Urease activities in 0–20 and 20–40 cm soil layers under different wheat straw incorporation treatments. Note: CK, no straw incorporation; L, incorporation of straw at a low rate of 3 000 kg hm^-2^ wheat straw; M, incorporation of straw at a medium rate of 6 000 kg hm^-2^ wheat straw; H, incorporation of straw at a high rate of 9 000 kg hm^-2^ wheat straw. Bars with different lowercase letters indicate significant differences (*P*<0.05).

After four years of straw incorporation practices, the urease activity levels in the 0–40 cm soil layers were higher with all treatments compared with CK (Fig. 1). With the increase amount of wheat straw incorporation, the invertase activity levels were 15.3%, 42.2% and 32.5% higher with the three treatments respectively than CK in 2011, only the H and M treatments were significantly superior to the control (P < 0.05).

#### 3.3.2 Soil invertase activity

**Fig 2:**
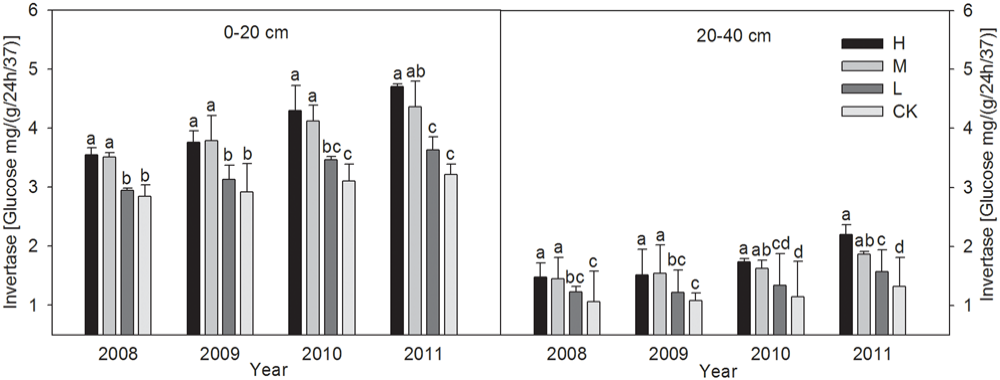
The changes of Invertase activities in 0–20 and 20–40 cm soil layers under different wheat straw incorporation treatments. Note: CK, no straw incorporation; L, incorporation of straw at a low rate of 3 000 kg hm^-2^ wheat straw; M, incorporation of straw at a medium rate of 6 000 kg hm^-2^ wheat straw; H, incorporation of straw at a high rate of 9 000 kg hm^-2^ wheat straw. Bars with different lowercase letters indicate significant differences (*P*<0.05).

After the harvest during each of the four years (Fig. 2), the soil invertase activity levels in the 0–40 cm layers with different treatments were higher than the control, the H and M treatments showed much more potential in increasing the soil invertase activity levels as the study years went on. And throughout the wheat straw incorporation experiment, the increase of soil invertase activity levels with the H treatment than the CK were in the range of 28.6–52.1% (P < 0.05), and the increase range of the M treatment were 26.9–37.3% (P < 0.05), whereas there were no significant differences between L and CK.

#### 3.3.3 Soil phosphatase activity

**Fig 3:**
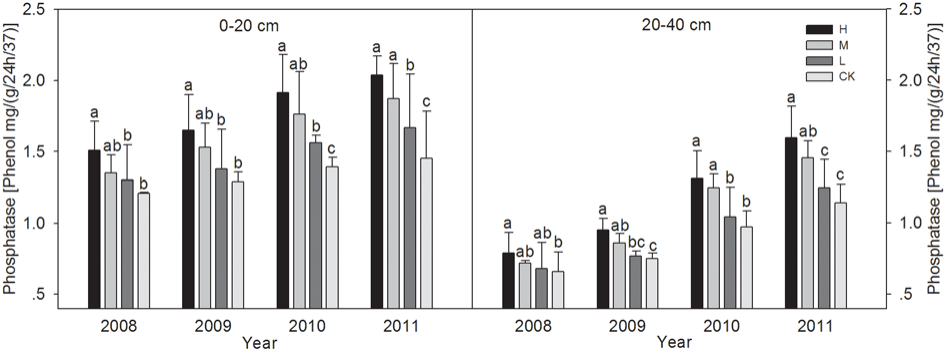
The changes of Phosphatase activities in 0–20 and 20–40 cm soil layers under different wheat straw incorporation treatments. Note: CK, no straw incorporation; L, incorporation of straw at a low rate of 3 000 kg hm^-2^ wheat straw; M, incorporation of straw at a medium rate of 6 000 kg hm^-2^ wheat straw; H, incorporation of straw at a high rate of 9 000 kg hm^-2^ wheat straw. Bars with different lowercase letters indicate significant differences (*P*<0.05).

Fig. 3 shows that with four years of wheat straw incorporation, the phosphatase activity levels in the 0–40 cm layers with all treatments have been improved to some degree, and different treatments were ranked in the order: M > H > L > CK. After the wheat harvest in 2011, the phosphatase activity levels in the 0–20 cm soil layers were 40.2% and 28.7% significantly higher with H and L than CK (P < 0.05), respectively. The H and M treatments resulted in 39.6% and 27.3% significantly higher phosphatase activity levels than CK (P < 0.05), respectively, whereas there were no significant differences between CK and L.

### 3.4 Grain yield and harvest index (HI)

During the study period, the crop yields with the straw incorporation treatments were significantly different from those with CK (Table 5). The grain yields with H, M, and L were higher than that with CK, i.e., 19.31%, 18.02%, and 6.84% higher in 2008; 26.60%, 22.40%, and 6.58% higher in 2009; 28.43%, 24.31%, and 7.94% higher in 2010; and 34.09%, 21.34%, and 7.32% higher in 2011, respectively. The grain yields agreed with the precipitation levels in each growing season.

**Table 5:** Grain yields and harvest indexes under different wheat straw incorporation treatments from 2008 to 2011.

Treatments	Grain yield (kg hm^-2^)				Harvest Index			
	2008	2009	2010	2011	2008	2009	2010	2011
H	4524a^1^	4868a	4743a	4118a	0.40a	0.39a	0.40a	0.39a
M	4475a	47076a	4591a	3726b	0.41a	0.39a	0.39a	0.38a
L	4051b	4099b	3986b	3295c	0.40a	0.38ab	0.38ab	0.37b
CK	3792c	3846c	3693c	3071d	0.40a	0.37b	0.38b	0.37b

Note: CK, no straw incorporation; L, incorporation of straw at a low rate of 3 000 kg hm^-2^ wheat straw; M, incorporation of straw at a medium rate of 6 000 kg hm^-2^ wheat straw; H, incorporation of straw at a high rate of 9 000 kg hm^-2^ wheat straw.

^1^ Values within a column for the same year followed by different letters are significantly different (*P*<0.05)

During 2008–2011, the mean harvest indexes (HI) with H, M and L treatments were increased by 5.21%, 4.41%, and 1.25% compared with CK, respectively. HI did not differ significantly between L and CK.

## Discussion

### 4.1 Available soil nutrients

As the basic substrate for crop growth, the soil nutrient contents are considered to be good indicators of the soil quality and productivity [28], and it is recognized that the available soil nutrients (including N, P, and K) derived from mineralization and the available components of fertilizer can be absorbed directly by plants, thereby contributing greatly to the soil fertility. Many studies have shown that the regular and appropriate addition of organic materials (crop residues) have significant effects on the physical and chemical properties of soil [13,29], which have essential roles in improving soil organic matter dynamics and nutrient cycling [30], as well as sustaining the rice productivity of soil [31]. Thus, the incorporation of crop residues into the soil and its subsequent decomposition replenishes the soil organic matter content, but it also supplies essential nutrients after mineralization (N, P, S, and Si) and even after soaking (K) [32], which affects the microbial population and the enzyme activity levels in the soil with subsequent transformation of nutrients.

The current study of wheat straw incorporation in a dryland farming region (Heyang) showed that, compared with the application of chemical fertilizer alone, straw incorporation had a highly significant effect on the available nutrient contents in the 0–40 cm soil layer, especially that in the top soil layer (0–20 cm). Similar to the results of the present study, a field experiment conducted by Pathak *et al*. [27] at the Indian Agricultural Research Institute during 2002–2003 showed that both the nutrient contents and their availability increased after the incorporation of crop residues in the plowing layer. Liu *et al*. [33] also showed that long-term straw retention and the application of chemical fertilizers in Pingliang, Gansu, China could increase the AP and AK concentrations in the top soil layer, whereas they were lower in the absence of straw. A 10-year study of the effects of various crop residue amounts on selected soil properties by Power *et al*. [34] showed that the return of increasing amounts of residues to a soil depth of 30 cm in a silty clay loam enhanced the soil AN and AP contents compared with NPK fertilizer alone. Zhang *et al*. [35] investigated the effects of chemical fertilizer and wheat straw incorporation on soil fertility in a spring wheat continuous cropping system on a Kastanozem soil in the Hehuang irrigation region of Qinghai province and showed that the soil N, P, and K contents were higher with straw incorporation treatments compared with chemical fertilization treatments, where the nutrient contents increased with the amount of straw. In the present study, the average increase in the AN contents of the 0–40 cm soil layers with straw incorporation treatments was 9.1–30.5%, with average AP and AK increases of 9.8–69.5% and 10.3–27.3%, respectively. These results agree with the findings of a five-year straw incorporation experiment in rice soil by Zhou *et al*. [36], who reported that N, P, and K contents increased substantially in the 0–20 cm soil layer. In particular, the AP content was 86.7–123.5% higher, the AN content was 68.1–81.9% higher, and the AK content increased the least. This may have been because the high moisture levels and temperatures in paddy fields promoted the decomposition of straw.

However, various studies have reached different conclusions about the effects of straw incorporation on the available soil nutrients. Based on a long-term analysis of a moist soil with low fertility, Lao *et al*. [37] found that the soil hydro-N content increased significantly with increasing amounts of straw incorporation into the soil, which may have been attributable to N supply from the crop residues and/or the improved physical and chemical condition of the soil, as reported by Utomo *et al*. [38]. Garg and Bahl [15] reported that the combined use of chemical fertilizers and organic manures or crop residues in Samana sandy loam and Ladhowal silt loam soils resulted in significantly higher P availability compared with chemical fertilizer treatment alone, which may have been due to the release of organic acids during decomposition, thereby supporting the release of P by solubilizing native P. However, this observation is not consistent with other studies. For example, Dong *et al*. [28] found that there were no obvious differences in the AN and AP levels between straw return and chemical fertilizer (NPK) treatments throughout the entire experimental period in the plowed layer (0–20 cm) of a paddy soil during 1998–2009 in Jiangxi Province, southern China, possibly because the levels of N and P in the residues were small compared with the total levels in the soil. According to Tan *et al*. [8], the levels of different forms of AK were high after NPK plus straw treatment in a Hebei fluvo-aquic soil and a Shanxi brown soil in northern China in 1992, whereas the levels were deficient after NPK treatment alone. It is possible that the level of K in the wheat straw comprised approximately 80% of that in the whole plant, most of which was returned to the soil, thereby increasing the K content [8]. Similar soil K enhancements with various crop residue amounts were reported based on experiments at the Directorate of Rice Research (DRR) farm, ICRISAT campus, India, which showed that the AK level increased significantly with 100% straw incorporation (about 377 kg hm^-2^) and with 50% straw incorporation (about 375 kg hm^-2^) compared with the control (355 kg hm^-2^) on a sandy clay loam soil [32]. However, some studies have shown that residue incorporation treatments had no significant effects on the soil K levels [38,39].

### 4.2 Soil carbon contents

The SOC content is a major resource that links the chemical, physical, and biological properties of soils [40]. Pinheiro *et al*. [41] showed that soil exposure by tillage and the lack of residue inputs led to a decline in aggregation and organic carbon, both of which made the soil susceptible to erosion. Many studies have shown that the incorporation of residues into the soil is one of the most important factors that affects soil structural development and SOC improvement [19,42,43]. The results obtained in our study indicate that the straw incorporation treatments increased the SOC levels significantly by 0.7–23.4%, thereby effectively mitigating reduced SOC accumulation in the agroecosystem due to intensive cropping [44]. Overall, the SOC contents with the H and M treatments were significantly different compared with the L treatment throughout the four experimental years, where the SOC level was increased greatly by the incorporation and decomposition of straw. The results of a similar field trial showed that 10 years of wheat and corn residue incorporation increased the SOC level in red soil by 25% [45]. The interannual variations of SOC content in our study showed a relatively stable trend, the probable explanation may be that the changes in SOC are generally insensitive to recent management practices, as these changes occur slowly and are relatively small compared to the vast background of SOC, especially for low amount straw incorporated in soil [46], similar results were also obtained by experiment on silty clay soil [5] and experiment on a sandy clay loam soil [32].

The LOC is a sensitive indicator for evaluating changes in the soil quality, because the LOC plays vital roles in nutrient cycling and microbial activity energy uptake [47]. In our study, the LOC contents tended to be greater in the 0–40 cm soil layers after crop residue incorporation compared with the control. This improvement may be related to the direct inputs of C via the leaching of dissolved organic C from crop residues into the soil [48], as well as by increased soil aggregation because the formation of soil aggregates protects the LOC from microbial decomposition [49]. Similar effects were reported by Shah *et al*. [50].

### 4.3 Soil enzyme activities

Soil enzymes are known to be related to microbial activity and they catalyze specific reactions and nutrient cycling in soils [51]. Soil enzymes have also been suggested as potential indicators of soil quality, which could integrate chemical, physical, and biological characteristics to monitor the effects of soil management on long-term productivity [52]. A number of studies have shown that residue incorporation in the soil can increase the activity levels of various soil enzymes [53,54], and the levels are usually higher than those after treatment with chemical fertilizers alone [33]. A 31-month field study conducted by Martens *et al*. [55] showed that addition of different organic residues (barley straw and fresh alfalfa) to an Arlington coarse-loamy soil greatly increased the activities of urease, invertase and dehydrogenase. And baley straw incorporation was the most effective amendment, which may be due largely to the enhanced humus content. Zhao *et al*. [56] conducted a 25-year fertilizer experiment to study the effects of wheat straw incorporation on soil properties and crop yields in a crop rotation system in semiarid conditions in China, which showed that the activity levels of invertase, urease and alkaline phosphatase in the topsoil (0–15 cm) were higher with straw manure combined with chemical fertilizer (straw + NP) compared with the control (NP).

Several trends in the enzyme activity levels were detected in our study. Compared with the control, the straw incorporation treatments greatly increased the activity levels of soil urease, alkaline phosphatase, and invertase in the 0–40 cm soil layers, where there were greater increases in the 0–20 cm layers than the 20–40 cm layer. This may have been because the microbial population and microbial biomass C or N were increased by straw incorporation, which provided organic matter that was used as a substrate for soil enzymes [55]. The crop residue itself may also have provided abundant organic matter, which supported the growth of micro-organisms [46]. The activity levels were higher in the topsoil, which may have been due to the “surface activation effect” [53]. Our analysis agrees with the findings of Dick *et al*. [4] who also reported that long-term residue treatments significantly affected the enzyme activities (alkaline phosphatase and urease) in the 0–20 cm soil layers of semiarid soils in the Pacific Northwest, which were possibly attributable to the higher levels of endoenzymes in the viable microbial populations and increased levels of accumulated soil enzymes in the soil matrix. In a 19-year study of various fertilization treatments on paddy soils at Changshu agroecological experiment station, Wang *et al*. [57] found that NPK fertilizer plus straw treatment caused significant increases (*P* < 0.05) in the urease and invertase activity levels (30.7% and 85.5%, respectively) compared with NPK fertilizer treatment alone. Similar results were also observed in different soil types, such as maize residue incorporation in clay soil [58] and wheat straw incorporation in sandy loam [2]. However, Marschner *et al*. [59] reached somewhat different conclusions, where the activities of most of the enzymes assayed in a calcareous Regosol soil in the Rhineland lignite mining area were not affected significantly by long-term crop residues, which may have been due to the relatively small amounts of organic amendments applied. The conflicting results and interpretations reported from large-scale studies may be related to different climatic and soil conditions, crop types, tillage regimes, and residue incorporation methods.

In the latter two years of the present study, the enzyme activity levels tended to increase with the straw incorporation amount, although the results did not differ significantly with the high and medium application levels. The results obtained in a long-term paddy soil fertility experiment in the Taihu lake region also indicated that straw incorporation treatments can play important roles in enhancing the activities of soil alkaline and acid phosphatases, dehydrogenase, and urease, all of which increased with the amount of crop residues applied [60].

### 4.4 Grain yields

The productivity of grain crops is affected significantly by water availability and the soil fertility [61], thus well-managed soils can support sustainable production and improved the crop yields. Tripathy and Singh [62] and Karami *et al*. [63] reported positive effects on the crop yield and soil productivity after crop straw application, which were attributed mainly to the improved soil quality. In our study, the crop yields and harvest indexes were higher with straw incorporation than conventional tillage (CK). Similar effects were reported by Zhu *et al*. [10] based on an 8-year field experiment in China.

In summary, the results of our trial showed that increasing amount of wheat straw incorporated into soil can improved the SOC levels and has a significant effect on the accumulation LOC contents and soil available nutrients (N, P, K) in semiarid areas, most studies under various climate and soil types reached the similar conclusions [30,33,50]. Regular and appropriate addition of crop residue have essential roles in improving the activity levels of soil enzymes that are important for nutrient cycling, as well as increasing crop productivity [2,63], which are in accord with the results obtained in our study under high and medium application levels.

## Conclusions

In this four-year field experiment, the incorporation of high and medium amounts of wheat straw had significant effects on increasing the soil N, P, and K, the AP levels, where the latter increased more than the AK and AN contents, especially in the 0–20 cm soil layers. There were similar trends in the SOC, LOC, and enzyme activity levels, and the incorporation treatments significantly increased the grain yields, all of which increased with the amount of crop residues applied. Compared with other treatments, the 9000 kg hm^-2^ straw incorporation treatment (H) was the most effective practice for improving the soil properties and fertility, which can be recommended for dryland farming areas as a crop residue management system to enhance both agricultural productivity and sustainability, and because the amount of straw of H treatment is higher than 100% straw incorporation, adding extra wheat straw from nearby farmland is necessary in present, and further studies are in progress.
